# Effect of Percutaneous Coronary Intervention on Left Ventricular Diastolic Function in Patients With Coronary Artery Disease

**DOI:** 10.5539/gjhs.v8n1p270

**Published:** 2015-06-25

**Authors:** Nahid Salehi, Mohammadreza Saidi, Alireza Rai, Farid Najafi, Seedmokhtar Javeedannejad, Mehran Babanejad, Hooman Tadbiri

**Affiliations:** 1 Department of Cardiology, School of Medicine, Kermanshah University of Medical Sciences, Kermanshah, Iran; 2 Epidemiology Department, School of Population Health, Kermanshah University of Medical Sciences, Kermanshah, Iran; 3 Medical Students Research Committee (MSRC), Faculty of Medicine, Iran University of Medical Sciences (IUMS), Tehran, Iran

**Keywords:** percutaneous coronary intervention, left ventricular diastolic function, echocardiography, Iran

## Abstract

**Background:**

There is considerable disagreement over the effects of percutaneous coronary intervention (PCI) on left ventricular diastolic function that has necessitated the investigation of diastolic indices. The present study was conducted to evaluate left ventricular diastolic function and its indices, three months after performing the PCI procedure in patients with coronary artery disease (CAD).

**Methods:**

In a quasi-experimental clinical trial study (before and after), 51 patients with CAD scheduled for elective PCI were investigated provided that their Ejection Fraction (EF) was > 30%. Before and three months after PCI, echocardiography was carried out to evaluate left ventricular diastolic indices including the E/Ea as the most important criteria for diagnosis of diastolic heart failure (DHF).

**Results:**

Based on the E/Ea indices and after PCI, the number of patients with DHF decreased significantly: 40 patients (78.4%) before PCI versus 28 patients (54.9%) after PCI (p<0.05). The Mean and Standard error of deceleration time (DT), isovolumic relaxation time (IVRT), early diastolic mitral annulus velocity; Ea (E’), E/Ea and left ventricular ejection function (LVEF) indices underwent significant changes. In addition, MVA dur/PVA dur, PVs/PVd, and E/Ea indices had changed significantly after PCI in both genders. However, no significant difference was reported for the other indices.

**Conclusion:**

The E/Ea ratio as an important criterion for diagnosis of DHF was improved after PCI. Improvement of several other diastolic indices was observed after the PCI procedure. It can be concluded that PCI can be an effective treatment modality in patients with left ventricular diastolic indices.

## 1. Introduction

Coronary Artery Disease (CAD) is one of the major causes of death in the world that kills many people annually (Rasmond, Flegal, & Friday, 2007; Williams, 2009). Frequency of coronary artery risk factors in Iran is, to a great extent, similar to the Mediterranean countries and even larger than the Western countries (Ebrahimi, Kazemi-Bajestani, Ghayour-Mobarhan, & Ferns, 2011). At least one of the conventional risk factors of coronary artery disease including hypertension, smoking, diabetes and obesity shows up in approximately 80% of Iranians aged 20 and over (Azizi, Rahmani, Emami, Mirmiran, Hajipour, Madjid et al., 2002).

PCI is a procedure performed to relieve ischemic symptoms in patients with CAD. The application of this method to prevent heart failure can be as effective and efficient as coronary artery bypass grafting (CABG). Coronary artery stenosis can result in a heart failure and consequently systolic and diastolic heart failure, in case it is disregarded (American Heart Association, 2002; R. O. Bonow, Mann, Zipes, & Libby, 2011; Serruys et al., 2001). Diastolic dysfunction occurs as a result of a variety of risk factors including diabetes, hypertension, and aging, comprising about 50 –55% of all cardiac dysfunctions. Most of the drug and nondrug therapy methods with well-documented efficacy in the treatment of systolic dysfunctions have been ineffective in curing diastolic heart failure or have had little clinical effects (R. O. Bonow et al., 2011; Redfield, Jacobsen, Burnett Jr, Mahoney, Bailey, & Rodeheffer, 2003; Zile & Brutsaert, 2002). On the other hand, the disagreement over the effects of PCI on systolic and diastolic functions of the heart makes it worthwhile analyzing diastolic heart indices after intervention (Buszman et al., 2007; Silva et al., 2005).

In a study conducted on patients with kidney dysfunctions, diastolic indices like Deceleration Time (DT) and E/Ea significantly decreased, while intervention had no impact on E/A and Isovolumic Relaxation Time (IVRT) (Namazi, Mohseni Badalabadi, Vallaei, Safi, Saadat, Vakili et al., 2010). In another study carried out on the coronary arterypatients, improvement was observed only for E/A and E’ indices with no significant changes in diastolic indices for other cases (Hashemi, Motamedi, Khani, Hekmat, Gachkar, & Rezaeefar, 2010). Furthermore, Tanaka et al have reported that the diastolic function of heart improved in patients with CAD after PCI (Tanaka et al., 2006).

Studies of the therapeutic effects of PCI in Iran have mostly focused on the systolic function of the heart and have rarely examined diastolic function because the former is easier to use. A few others that exist are simply investigations of the overall performance of left ventricular diastolic function with no consideration of the patients’ precise cardiographic findings (Nozari, Oskouei, & Khazaeipour, 2012). Thus, given the high incidence of left ventricular diastolic failure in patients with CAD and the possible development of cardiac dysfunction, and, because some echocardiography indices such as E/Ea ratio can be amongst the best criteria for DHF diagnosis, there seems to be a necessity for conducting a study to analyze the role of PCI in improving diastolic function of the heart based on echocardiography findings. Therefore, the present study was conducted to evaluate left ventricular diastolic function three months after PCI procedure in patients with CAD.

## 2. Methods

### 2.1 Data Source and Study Population

The present study was a quasi-experimental clinical trial study (before and after) conducted in Imam Ali educational research center (Imam Ali hospital is a referral center in the west of Iran located in Kermanshah province, which covers the 2 million population within the province and patients referred from neighboring provinces).

### 2.2 Sample Size

Based on a study conducted in Iran and the rates of IVRT, Mitral EV and Mitral AV indices, the maximum sample size was estimated for 51 patients considering the changes in Mitral EV (Power 80%, d= 0.02, standard deviation=0.05) (Hashemi et al., 2010). Patients with CAD with an EF over 30% and traces of diastolic dysfunction were considered for the study.

### 2.3 Exclusion Criteria

Exclusion criteria were well-known factors of left ventricular diastolic dysfunctions, including high blood pressure (over 140/90 mmHg), hypertrophic cardiomyopathy, left ventricular hypertrophy, high creatinine, bundle branch block, ventricular fibrillation, ventricular arrhythmia, severe valvular disease, complete heart block and previous CABG. The participants underwent left ventricular diastolic analysis using echocardiography according to the guidelines of American Echocardiography Association before PCI was performed.

### 2.4 Study Variables

IVRT (ms), E/A Ratio of Mitral Inflow Velocity (E/A ratio), MVA duration, PVA duration, Pulmonary vein systolic flow/Pulmonary vein diastolic flow (PVs/PVd), Deceleration Time (DT) and Ea (E’) parameters were determined using Tissue Doppler Imaging (TDI). E/Ea and Left Ventricular Ejection Fraction (LVEF) indices were also determined (Table 1). Diastolic Heart Failure (DHF) was compared before and after intervention. Accordingly, E/Earatio was considered as the diagnostic criterion for diastolic heart failure; ≤8 as healthy and >8 as DHF (16). Demographic and risk factor profile including age, sex, smoking history, hypertension history and drug consumption, high blood lipid, and diabetes were noted. All patients underwent PCI therapy and their echocardiographic indices were determined again after three months and after consuming the medications. The effects of PCI on the diastolic performance of the heart were evaluated by comparing the statistical data obtained through echocardiography and determining the differences in left ventricular diastolic performance in both the stages mentioned.

**Table 1:** The definitions and normal ranges of study variables

Variables	Definition	Normal
DT(msec)	Time taken from the maximum E point to baseline	160-240
IVRT(msec)	The time interval between aortic valve closure and mitral valve opening	70-90
E/A ratio	The ratio of the early (E) to late (A) ventricular filling velocities	1-1.5
MVAdur/PVA dur	The ratio of the late ventricular filling duration (A) to the pulmonary reversal flow duration (A)	≥ 1
PVs/PVd	The ratio of pulmonary vein systolic flow velocity to the pulmonary vein diastolic flow	≥ 1
Ea (E’) (cm/sec )	Peak velocity of tissue Doppler velocity of septal mitral annulus	> 8
E/Ea ratio	The ratio of the early ventricular filling velocity (E) to the peak velocity of tissue Doppler velocity of septal mitral annulus	≤ 8 normal >8 showing DHF
LVEF %	Left ventricular ejection fraction percentage	≥ 50

### 2.5 Ethics

The study received the approval from the ethical committee of Kermanshah University of Medical Sciences. Before the study, all patients signed the consent form to participate in the study, and thereafter PCI was performed.

### 2.6 Statistical Analysis

The obtained data were fed into SPSS software (version 16) and analyzed by Fisher test, paired sample t-test and independent t-test. p <0.05 was considered significant.

## 3. Results

The study included 51 patients. Thirty-six (70.6%) patients were males and the rest were females. The mean and standard deviation obtained with the age range of 40–77 was 59.61±8.76. In terms of risk factors, 30 (58.8%) patients had high blood pressure followed by 19 (37.3%) patients with high blood lipid. Smokers comprised 25.5% of the total (13 patients) and diabetics accounted for 17.6% of the population (9 patients). The results of the angiography carried out before PCI indicated that 27 (52.9%) had one-vessel, 17 (33.3%) had two-vessel, and 7 (13.8%) had three-vessel coronary artery disease.

As for DHF and taking the E/Ea ratio into account, 11 (21.6%) of the participants were healthy but 40 (78.4%) of them were sick before the intervention. However, the number of healthy people almost doubled to 23 (45.1%) and the number of sick patients dropped to 28 (54.9%), (p=0.014), (Table 2).

**Table 2:** Changes of Diastolic Heart Failure (DHF) before and after PCI

	Before PCI, number (%)	After PCI, number (%)	P value
Normal	11 (21.6)	23 (45.1)	
Diastolic Heart Failure (DHF)	40 (78.4)	28 (54.9)	0.014
Total	51 (100.0)	51 (100.0)	

Among the indices of diastolic performance of the heart, the mean and standard error for DT, IVRT, Ea (E’), E/Ea, and LVEF indicated significant change after PCI followed by medication. However, E/A, MVA dur/PVA dur, and PVs/PVd remained unchanged. The pattern of these changes showed a decline for DT, IVRT, and E/Ea indices and an increase for the other indices (Table 3).

**Table 3:** Changes of diastolic heart performance after PCI

Variable	Before PCI	After PCI	Difference	P value	Status
DT(msec)	224.24±7.58	192.33±5.9	-31.9±7.55	< 0.001	Improved
IVRT(msec)	104.25±3.19	85.49±1.86	-18.76±3.62	<0.001	Improved
E/A Ratio	0.83±0.04	0.85±0.03	0.02±0.04	0.64	Almost improved
MVA dur/PVA dur	1.13±0.03	1.19±0.03	0.06±0.04	0.15	Worse
PVs/PVd	1.44±0.05	1.56±0.04	0.11±0.06	0.09	Almost improved
Ea (E’)	6.12±0.15	7.49±0.21	1.37±0.2	> 0.001	Improved
E/Ea Ratio	11.19±0.52	9.3±0.37	-1.89±0.44	< 0.001	Improved
LVEF %	47.59±0.97	49.36±1.08	1.76±0.63	< 0.01	Improved

*Mean and standard Error.

After PCI, the mean and standard errors of MVA dur/PVA dur, PVs/PVd, and E/Ea indices changed significantly in both genders; MVA dur/PVA dur increased in male and PVs/PVd increased in both genders. In contrast, E/Ea index decreased in both males and females (p<0.05). For DT, IVRT, E/A, Ea(E’), and IVEF, however, there was no statistically significant difference in terms of gender. DT and IVRT decreased in both genders. Other indices (except E/A) showed an increase in males (p>0.05) (Table 4).

**Table 4:** Correlation of diastolic heart indices and gender before and after PCI

Variable	Before PCI		P value	After PCI		P value
	
	Male	Female		Male	Female	
DT(msec)	200.27±15.13	234.22±8.28	0.04	187.4±13.08	194.39±6.43	0.59
IVRT(msec)	98.27±7.41	106.75±3.3	0.23	81.87±3.81	87.00±2.08	0.21
E/A Ratio	0.8±0.09	0.84±0.05	0.72	0.89±0.09	0.83±0.03	0.51
MVAdur/PVA dur	1.09±0.06	1.15±0.04	0.47	1.09±0.06	1.23±0.03	0.04
PVs/PVd	1.47±0.1	1.44±0.06	0.79	1.72±0.08	1.49±0.05	0.01
Ea (E’)	6.07±0.34	6.14±0.17	0.83	7.27±0.34	7.58±0.27	0.5
E/Ea Ratio	13.26±1.19	10.33±0.49	0.009	10.69±0.75	8.72±0.39	0.01
LVEF %	49.83±1.27	46.66±1.24	0.13	51.33±1.37	48.54±1.42	0.24

## 4. Discussion

DHF has been reported in over 35–42% of the patients with heart failure symptoms (Betocchi & Hess, 2000; Vasan, Benjamin, & Levy, 1995). DHF has been reported in over 35–42% of the patients with heart failure symptoms However, 30–40% of the patients with heart failure, despite having normal EF, suffered from clinical heart failure symptoms. In fact, these patients like those with low EF level are exposed to risk factors and heart failure (Brogan III, Hillis, Flores, & Lange, 1992). That is why, only patients with EF over 30 were investigated. It is important to note however that the diagnostic criteria for diastolic heart failure have not been definitively established.

According to Kasner et al. (2007), E/Ea ratio can be one of the best criteria for DHF diagnosis; indices ≤8 indicating healthy and those >8 signifying DHF (Kasner, Westermann, Steendijk, Gaub, Wilkenshoff, Weitmann et al., 2007). The findings of the present study indicated a significant decrease forE/Ea index after intervention, and the improving trend was indicative of the influential diagnostic role of this method in patients with DHF. Other studies have also indicated the ameliorating effect or decreasing E/Ea level after PCI in patients with CAD (R. Bonow, Kent, Rosing, Lipson, Bacharach, Green et al., 1982; Hashemi et al., 2010).

The diastolic performance indices were analyzed three months after PCI. The findings demonstrated a decrease in the mean and standard error of DT, IVRT, and E/Ea indices. E/A, MVA dur/PVA dur and PVs/PVd levels did not show any significant changes. Inan analysis of the effect of angioplasty or PCI on the diastolic performance of patients with renal insufficiency, Namazi et al, (2010) have not demonstrated a significant impact of PCI on the E/A index, which is in line with the findings of the present study. They also indicated a decline for DT and E/Ea indices. Although they did not report any significant change in the IVRT index, in the present study, it decreased significantly. This difference may be justified bya longer reevaluation time in which their patients were investigated six months after PCI in comparison with the three-month period of the present study (Namazi et al., 2010). Contrary to our results, in another study, no significant changes were observed in DT and IVRT levels three months after PCI in the patients, but these indices had a declining trend. They noted a significant decline in E/A ratio after intervention, which is not compatible with the results of the present study (Hashemi et al., 2010).

According to our findings, Ea (E’) and IVEF levels increased after intervention. In the studies conducted by Surucu et al and Hashemi et al. (2010) a significant increase was also reported for E’ levels following PCI, which is in line with the results of the present study (Hashemi et al., 2010; Sürücü, Tatlı, Okudan, & Aktoz, 2009).

Moreover, after PCI, Beitnes et al. have concluded that LVEF as a diastolic index increased in the first three months and then decreased in the second three months and the following six months. However, this decrease was not statistically significant, which probably confirms the effect of time on diastolic indices after PCI (Beitnes et al., 2011).

The limitation of this study is proving an association in a quasi-experimental study of this nature with an n=51 (small sample size) and this should be considered in future studies with a larger sample size.

## 5. Conclusion

Among the variables analyzed after intervention, DT, IVRT, Ea (E’), E/Ea Ratio and LVEF indices showed an improvement. The E/Ea index, thought to be the main criterion to determine DHF was also improved. From the findings of the present study, it can be argued that PCI in patients with CAD can ameliorate the compromise in diastolic heart function. As an effective step in clinical management, decreasing the E/Ea index using the PCI method in patients with CAD may decrease the chance of DHF

The role of confounding factors including medication use before & after PCI, high mean age of the participants, smoking, hypertension, diabetes and high blood lipid that can affect diastolic performance after PCI is important and needs to be considered in the future studies. Furthermore, future studies are advised to employ larger study samples and longer follow-up periods, which help eliminate the effects of confounding factors to investigate the symptoms in different patient groups according to the ejection fraction (EF).
